# Infected Pseudoaneurysm of the Radial Artery

**DOI:** 10.1055/a-2039-3563

**Published:** 2023-05-29

**Authors:** Haruki Mizuta, Heishiro Fujikawa, Hisashi Motomura

**Affiliations:** 1 Department of Plastic and Reconstructive Surgery, Graduate School of Medicine, Osaka Metropolitan University, Osaka, Japan

The incidence rates of pseudoaneurysms of the radial artery, local infections, and bacteremia as complications of arterial cannulation procedures are relatively low, affecting only 0.048, 0.4, and 0.6% patients, respectively. 1 2 The current report describes the successful treatment of an infected pseudoaneurysm of the radial artery, an extremely rare complication of arterial cannulation, 3 by early surgical intervention.

A 65-year-old male patient was diagnosed with cardiac arrest caused by anaphylactic shock after undergoing contrast-enhanced computed tomography (CT). The patient received intensive care and underwent careful examination for autoimmune polyneuropathy. His previous medical history included renal transplantation for gouty nephropathy.

**Fig. 1:**
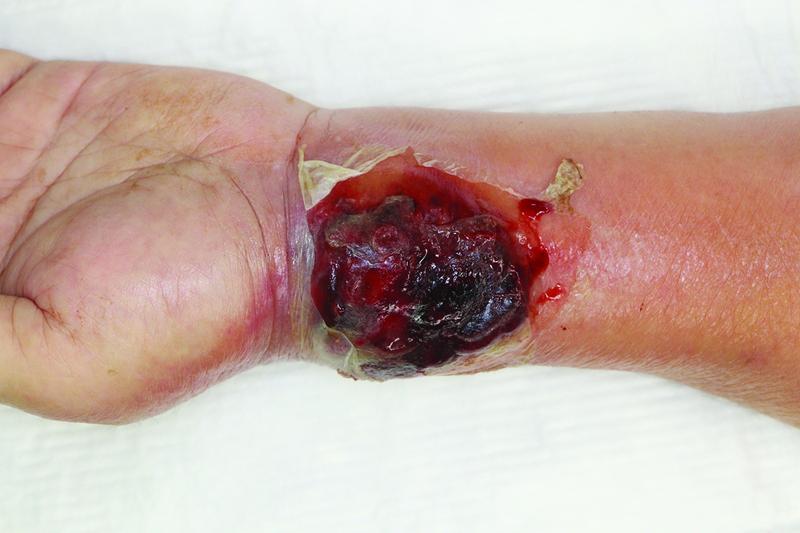
Clinical examination observed at the time of initial presentation of the patient at our department showed a dark-red tumor with blisters and abnormal pulsation on the left wrist.

Twenty-five days later, the patient exhibited progressive erythema at the site of radial artery cannulation and, upon suspecting cellulitis, intravenous administration of cefazolin and vancomycin was initiated. Blood culture examination revealed methicillin-resistant *Staphylococcus aureus* , and antibiotic treatment was commenced. The affected area was seen to be markedly swollen, blistered, and exhibited visibly abnormal pulsations 3 days after commencement of treatment ( Fig. 1 ).

**Fig. 2:**
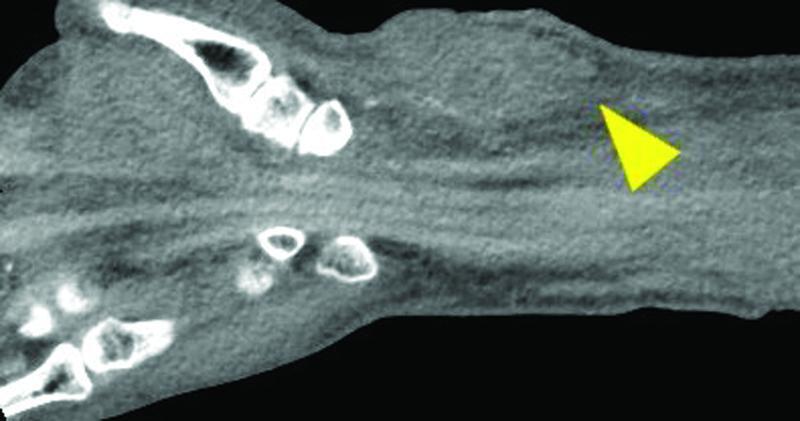
Clinical imaging (computed tomography [CT]): CT revealed low-echoic lesion at his left wrist, suggesting an infected pseudoaneurysm (yellow arrowhead).

**Fig. 3:**
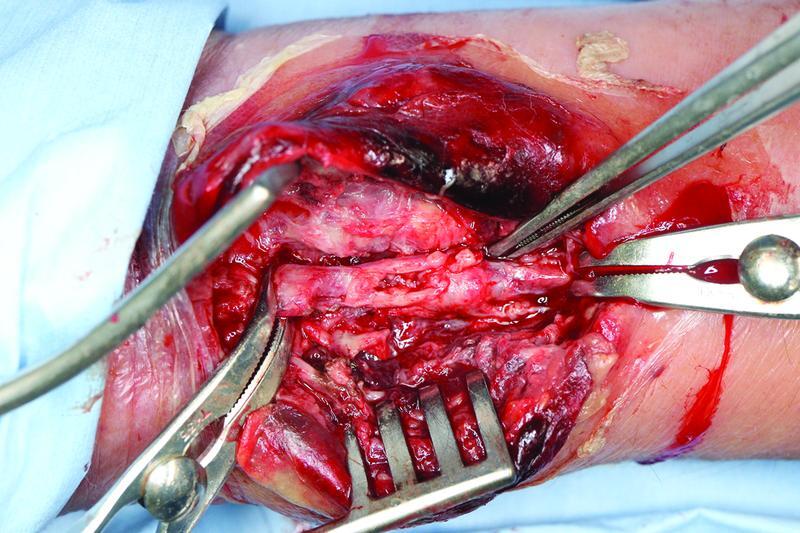
Partially ruptured radial artery was observed intraoperatively, and arterial ligation was performed immediately.

As the patient had a history of chronic renal failure, a CT scan was performed which revealed an infected pseudoaneurysm ( Fig. 2 ), necessitating emergency surgery. The preoperative Allen test was found to be negative and, following surgical debridement, the radial artery was ligated and the pseudoaneurysm was excised. The radial artery was seen to be partially ruptured ( Fig. 3 ). The radial and median nerves were preserved, and negative pressure wound therapy (NPWT) (V.A.C., KCI Co., Tokyo, Japan) was carried out.

We started the administration of NPWT at a pressure of –75 mm Hg in a continuous mode. Four days after NPWT, granulation appeared gradually. However, palmaris longus and flexor carpi radialis tendon remained exposed. We increased the pressure to –100 mm Hg. Using basic fibroblast growth factor (bFGF, Fiblast spray), we applied the OASIS collagen matrix (Cook Biotech Incorporated, United States) to tendon-exposed areas. The conditions were favorable for enhanced granulation; therefore, we increased the pressure to –125 mm Hg and modified the pressure every 3 days. Three weeks after NPWT, tendon-exposed areas showed complete granulation; thus, we considered this as the time required to complete NPWT.

**Fig. 4:**
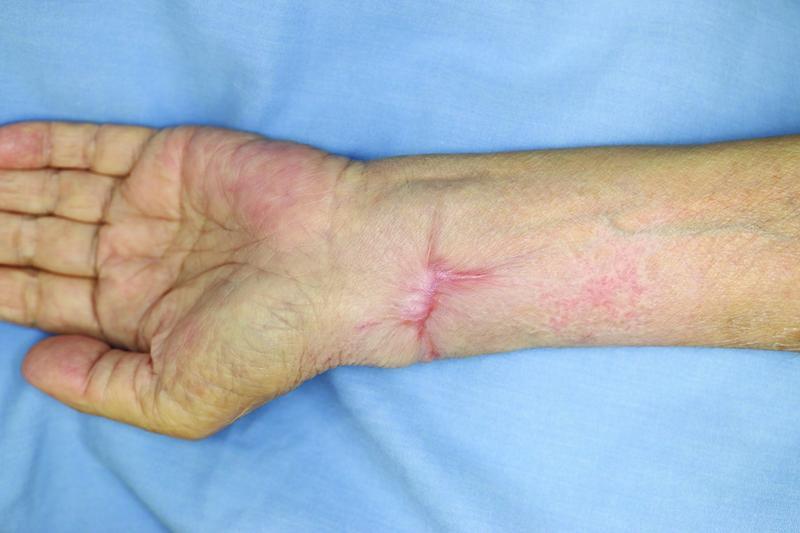
Clinical observation after 6 months showed complete wound healing.

The patient exhibited complete wound healing after 2 months, and no complications other than a slight sensory disorder of the dorsal thumb was observed ( Fig. 4 ).

*Staphylococcus aureus* are the most commonly observed etiological agent in infected pseudoaneurysms of the radial artery. 4 Risk factors for the development of infections associated with radial artery catheters include prolonged catheterization (> 4 days) and hospitalization, 2 while *Staphylococcus aureus* infections, persistent bacteremia for at least 48 hours after catheter removal, and antibiotic therapy have been shown to affect prognosis. 4 Conditions of both our cases are matched. 5

Infected pseudoaneurysms are clinically characterized by rapidly swelling wine-red tumors with pulsation. Diagnosis can be made based on clinical observation of a lesion that shrinks upon compression and rapidly refills when released, recognition of an aneurysm through CT or ultrasound examination, and detection of bacteria using blood culture examination. 4

However, not all cases may present with the typical symptoms. Some patients (including that presented in the current case report) do not exhibit pulsation in the early stages of the disease, making it difficult to distinguish the lesion from cellulitis caused by arterial cannulation.

Infected pseudoaneurysms of the radial artery have a high risk of rupture and require immediate arterial ligation. In case of ischemia in the peripheral region caused by ligation of the radial artery, revascularization using a vein graft should be considered. Therefore, an Allen test should also be performed preoperatively in all cases.

Noninfectious pseudoaneurysms that are small in size (< 3 cm), stable, and asymptomatic often thrombose within 4 weeks, and follow-up can be recommended in these cases. 3 However, this is only applicable for noninfectious pseudoaneurysms that occur after catheter removal or trauma, and those occurring during indwelling of a radial artery catheter require more careful management.

Previous evidence suggests that approximately 40% of the patients with local infections caused by radial artery catheterization went on to develop a pseudoaneurysm, 2 suggesting that these lesions can be expected to evolve into infected pseudoaneurysms, even in the absence of an infection initially. 5

Relatively early diagnosis, timely surgical intervention, careful wound management using NPWT, and adequate debridement contributed to postoperative success in our patient.

To the best of our knowledge, there are a limited number of case reports focusing on infected pseudoaneurysms of the radial artery in the field of plastic surgery. The findings of this study suggest that these lesions require careful postoperative wound management and should, therefore, be given due importance by plastic surgeons.
